# Severe Hyponatremia due to Phlegmonous Trismus

**DOI:** 10.1155/2014/320438

**Published:** 2014-12-25

**Authors:** Yoshihiro Momota, Tomio Iseki, Tadashi Ohkubo

**Affiliations:** ^1^Division of Shikoku Kochi Office, Ministry of Health, Labour and Welfare, 1-1-3 Honmachi, Kochi 780-0870, Japan; ^2^Department of Anesthesiology, Osaka Dental University, 1-5-17 Otemae, Chuo-ku, Osaka 540-0008, Japan; ^3^First Department of Oral and Maxillofacial Surgery, Osaka Dental University, 1-5-17 Otemae, Chuo-ku, Osaka 540-0008, Japan; ^4^Department of Internal Medicine, Osaka Dental University, 1-5-17 Otemae, Chuo-ku, Osaka 540-0008, Japan

## Abstract

We describe a patient with dysphagia and trismus associated with lower jaw inflammation due to phlegmon who developed severe hyponatremia from water intoxication due to excessive water intake after diaphoresis caused by abnormally hot weather. A 63-year-old woman presented with severe swelling of the floor of the mouth and trismus. As she had spasms and numbness of the extremities and restlessness and water intoxication caused by excessive water intake was suspected, she was hospitalized for the treatment of inflammation and electrolyte disorder. Although swelling of the floor of the mouth subsided over time after antimicrobial therapy, vomiting, diarrhea, and numbness of the extremities continued. On day 5 of hospitalization, severe vomiting and diarrhea recurred, and serum sodium levels decreased to 108 mEq/L. Decrease in water intake is essential in the treatment of hyponatremia. However, in patients with severe vomiting and diarrhea who can swallow only liquids because of hot weather and eating disorder, the risk of sodium depletion is high. It is important to restore electrolyte balance and fluid volume through supplementation with sodium, chlorine, potassium, and glucose among others, the reduction of intravenous fluid volume, and diuresis in order to correct the sodium level slowly.

## 1. Introduction

Phlegmon especially Ludwig's angina, also known as submandibular space infection, is a severe purulent inflammatory disease often associated with dental caries and may cause airway narrowing or obstruction, trismus, or dysphagia when inflammation spreads [1]. When severe trismus occurs, patients may not be able to eat solid food and tend to drink an excessive amount of water. As Japan is experiencing high temperatures in summer due to global warming, elderly people may suffer from dehydration even when they are at rest in their home.

We experienced a patient with trismus associated with oral infection who developed water intoxication after excessive water intake to prevent dehydration during very hot days, which resulted in serious hyponatremia due to excessive perspiration, vomiting, and diarrhea.

## 2. Case Report

A 63-year-old female patient with a history of total hysterectomy for the treatment of uterine rupture 30 years previously presented with a fever of 38°C that had lasted for 3 days and swelling of the right cheek and lower jaw. Antimicrobial treatment prescribed by a general practitioner did not relieve the swelling. She was unable to eat solid food because of odynophagia and trismus and had repeated episodes of vomiting and diarrhea due to excessive water intake during hot days with the highest temperature of ≥35°C. Physical examination at presentation revealed swelling and warmth in the submandibular and buccal regions and mild airway narrowing. She complained of numbness of the extremities and cramps in the calves, hyperpnea, and restlessness. Blood pressure was 115/84 mmHg, pulse 72 bpm, arterial oxygen saturation 99%, and blood glucose 98 mg/dL. Since serum biochemistry and hematology examinations revealed abnormal electrolyte values, inflammatory response, and anemia, she was hospitalized for drainage and fluid therapy. Under local anesthesia, an incision was made in the gingiva on the buccal side of the mandibular premolars, and a gauze drain was inserted in the incision. Since spasm and numbness of the extremities continued, she was treated with diazepam 2.5 mg. Fluid therapy was initiated with 5% glucose solution 250 mL and a hypotonic electrolyte solution 1,000 mL/day. Laboratory findings on day 1 of hospitalization were Na 117 mEq/L, K 3.5 mEq/L, and Cl 83 mEq/L. Since abnormal electrolyte levels, nausea, vomiting, and diarrhea continued, she received metoclopramide 10 mg intravenously and also received infusions of a hypotonic electrolyte solution 1,500 mL/day and physiological saline 700 mL/day to correct serum sodium levels. On day 2 of hospitalization, numbness of the extremities disappeared and nausea and vomiting improved, but watery stools continued. Although the distance she was able to open the mouth improved to about two-finger width and she became able to eat meals, she still complained of anorexia. Fluid therapy was continued with a hypotonic electrolyte solution 1,000 mL, maltose-lactated Ringer's solution 500 mL/day, and physiological saline 1200 mL. In the evening of day 3, electrolyte levels returned to normal ranges although inflammatory reaction was still observed. Fluid therapy was switched to physiological saline 100 mL on day 4. Since severe vomiting and diarrhea recurred at night on day 4 of hospitalization, she received metoclopramide 10 mg intravenously, and electrolyte infusion of a hypotonic electrolyte solution 1,000 mL, maltose-lactated Ringer's solution 500 mL, and physiological saline 200 mL was restarted. On day 6, as swelling in the buccal and mandibular regions subsided, the gauze drain was removed. However, generalized malaise, vomiting, diarrhea, anorexia, and numbness of the extremities recurred, and serious hyponatremia with Na 111 mEq/L, K 3.7 mEq/L, and Cl 79 mEq/L developed. She was treated with water restriction and infusion of maltose-lactated Ringer's solution 500 mL/day, a hypotonic solution 500 mL/day, and physiological saline 600 mL/day. On day 7, sodium and potassium levels were further decreased. She received physiological saline 100 mL, 20% glucose solution 20 mL, 10% NaCl 20 mL, maltose-lactated Ringer's solution mixed with one ample of potassium aspartate, and a hypotonic solution to correct hyponatremia and received furosemide 20 mg to induce diuresis. The urinary sodium level was within the normal range at 66.8 mEq/L. On day 8, serum and urinary sodium concentrations were maintained within the normal range by slow infusion with physiological saline 500 mL, 10% NaCl solution 40 mL, and maltose-lactated Ringer's solution mixed with one ample of potassium aspartate and diuresis, and vomiting and diarrhea disappeared. On day 9, electrolyte levels had been corrected with a CRP level of 0.59; fluid therapy was discontinued. On day 10, patient was discharged from the hospital after a full recovery (Table 1).

We obtained patient consent for publication of personal information.

## 3. Discussion

Submandibular cellulitis especially Ludwig's angina may induce airway obstruction [2–5], and when the patient is unable to open the mouth and eat enough meals, fluid imbalance and dehydration may develop. Patients with dehydration due to inadequate water intake often show increases in serum and urinary sodium levels and should be sufficiently hydrated and treated with hypotonic intravenous fluids, whenever necessary. The patient reported here could not open her mouth by more than 15 mm and had drunk a large amount of water during very hot days. As she complained of numbness of the extremities and leg cramps, water intoxication due to severe hyponatremia was strongly suspected.

Hyponatremia represents a relative or absolute increase in water content in the body. Clinically, the causes of hyponatremia include (1) excessive water intake and increase in water content in the body due to abnormal secretion/activity of antidiuretic hormone (ADH); (2) extrarenal sodium depletion through vomiting, diarrhea, or diaphoresis; and (3) increase in reabsorption of water, such as in edema, cirrhosis, and cardiac insufficiency [6]. In healthy individuals with normal osmolar and volume regulatory mechanisms, development of hyponatremia due to excessive water intake is unlikely since excess water can be excreted through osmotic diuresis. However, in patients with renal failure or syndrome of inappropriate secretion of antidiuretic hormone, hyponatremia due to inappropriate urinary dilution may develop [7]. In the present patient, we have no urine volume data and urinary sodium levels from before the onset of hyponatremia, and we thus cannot determine whether her hypotonic dehydration was caused by extrarenal sodium loss or renal sodium loss. However, as she had no present or past history of renal disorder, diabetes, or hyperlipidemia, the cause of hyponatremia in this patient was considered extrarenal sodium loss due to excessive perspiration, vomiting, and diarrhea and excessive water intake.

The present patient had numbness of the extremities and leg cramps but did not show central nervous symptoms. Severe or acute hyponatremia may be associated with headache, nausea, vomiting, lethargy, malaise, and decreased deep tendon reflexes and may cause brain edema and brain compression due to fluid overload. An abrupt decrease in sodium levels may also induce coma, convulsion, or respiratory arrest [7]. On the other hand, in patients with hyponatremia more than 48 hours after onset, intracellular osmolar pressure is already as low as that in extracellular fluid as the result of compensatory mechanisms. Abrupt supplementation of sodium may cause intracellular dehydration, which may lead to osmotic demyelination syndrome [8]. In the treatment of hypotonic hyponatremia, it is essential to correctly determine the severity of hyponatremia and whether the condition is acute or chronic.

Although it is unknown when serum sodium levels began to decrease in our patient, she had taken an excessive amount of water without solid meals for at least 48 hours before visiting the clinic. We, therefore, decided to treat her with restriction of oral water intake and supplementation with sodium to compensate extrarenal sodium loss due to vomiting and diarrhea. With fluid therapy with physiological saline at 700 mL/day and then physiological saline 200 mL/day and a hypotonic solution 1,500 mL/day, the serum sodium level was normalized by day 3 of hospitalization. Trismus improved with antimicrobial therapy, but diarrhea persisted. On the night of day 4, severe vomiting and watery stools recurred, and severe hyponatremia developed on day 7 of hospitalization. Her sodium balance was corrected by day 9 of hospitalization through maintaining normal urinary sodium levels with continuous intravenous infusion of a hypotonic solution, physiological saline, 10% NaCl solution, and a diuretic.

It has been recently reported that hyponatremia in association with changes in body temperature, perspiration, or respiration develops among people during marathons or other heavy exercise. Hyponatremia in this population is believed to be caused by water overload through excessive water intake regardless of thirst to avoid dehydration and increased secretion of ADH due to stress during the race [9]. In the present patient, it is considered that an increase in ADH secretion due to mental stress associated with inflammatory pain and trismus may have caused a decrease in urine volume, vomiting, and diarrhea, and excessive water intake to avoid dehydration during hot days caused water intoxication and hyponatremia.

Airway management is essential in the treatment of trismus and dysphagia associated with phlegmon. During summer, patients with mental stress due to pain and trismus may drink an excessive amount of water obsessively to avoid dehydration, which may cause water intoxication. Appropriate fluid management is essential to correct electrolyte abnormality.

## Figures and Tables

**Table 1:** Serum chemistry findings and electrolyte levels during hospitalization.

Normal range	Na 136–147 (mEq/L)	K 136–147 (mEq/L)	Cl 136–147 (mEq/L)	TP 6.7–8.3 (g/dL)	BUN 8–20 (mg/dL)	Cre 0.44–0.75 (mg/dL)	CRP <0.3 (mg/dL)	U-Na 40–160 (mEq/L)	Glu 70–110 (mg/d/L)	Infusion of electrolytes and glucose	Administration of drugs
Admission (day 0)	*116 *	*3.3 *	*83 *	*5.9 *	14.7	0.58	16.8			5% glucose 250 mL + hypotonic solution 1000 mL	Diazepam 2.5 mg

Day 1	117	3.5	83	6.0	7.8	0.48	12.9		113	Hypotonic solution 1500 mL + saline 700 mL	Aspoxicillin 6 g + metoclopramide 10 mg

Day 2										5% maltose-lactated Ringer's solution 500 mL + hypotonic solution 1000 mL + saline 200 mL	Aspoxicillin 2 g

Day 3	138	3.7	102	6.8	5.5	0.59	5.13		81	5% maltose-lactated Ringer's solution 500 mL + saline 1000 mL	Aspoxicillin 4 g

Day 4										Saline 100 mL	Aspoxicillin 2 g + loperamide 1 mg

Day 5										5% maltose-lactated Ringer's solution 500 mL + hypotonic solution 1000 mL + saline 200 mL	Aspoxicillin 2 g + metoclopramide 10 mg

Day 6	*111 *	*3.7 *	*79 *	*6.1 *	5.3	0.38	1.11		74	5% maltose-lactated Ringer's solution 500 mL + hypotonic solution 500 mL + saline 600 mL	Cefmetazole 2 g + metoclopramide 10 mg

Day 7	108	3.0	75	5.7	2.9	0.36	0.80	66.8	52	5% maltose-lactated Ringer's solution 500 mL + hypotonic solution 500 mL + saline 100 mL + 10% NaCl 20 mL	Cefmetazole 2 g + 20% glucose 20 mL+ furosemide 20 mg + potassium L-aspartate 1A

Day 8	117	3.4	85				0.77	70		5% maltose-lactated Ringer's solution 500 mL + saline 500 mL + 10% NaCl 40 mL	Furosemide 10 mg + potassium L-aspartate 1A

Day 9	133	3.4	100				0.59				

Day 10	134	3.7	101	6.3	5.0	0.45			66		

TP, total protein; BUN, blood urea nitrogen; Cre, creatinine; CRP, C-reactive protein; U-Na, urine Na; Glu, glucose.
